# Achieving Adequate Margins in Ameloblastoma Resection: The Role for Intra-Operative Specimen Imaging. Clinical Report and Systematic Review

**DOI:** 10.1371/journal.pone.0047897

**Published:** 2012-10-19

**Authors:** Inoka De Silva, Warren M. Rozen, Anand Ramakrishnan, Mansoor Mirkazemi, Charles Baillieu, Ronnie Ptasznik, James Leong

**Affiliations:** 1 Department of Plastic and Reconstructive Surgery, Dandenong Hospital, Southern Health, David St, Dandenong, Victoria, Australia; 2 Department of Surgery, Faculty of Medicine, Monash University, Clayton, Victoria, Australia; 3 Department of Radiology, Dandenong Hospital, Southern Health, David St, Dandenong, Victoria, Australia; The University of Chicago, United States of America

## Abstract

**Background:**

Ameloblastoma is a locally aggressive odontogenic neoplasm. With local recurrence rates reaching 90%, only completeness of excision can facilitate cure. Surgical clearance has widely been based on pre-operative imaging to guide operative excision margins, however use of intra-operative specimen x-ray or frozen-section has been sought to improve clearance rates, and advanced imaging technologies in this role have been proposed. This manuscript aims to quantify the evidence for evaluating intra-operative resection margins and present the current standard in this role.

**Method:**

The current study comprises the first reported comparison of imaging modalities for assessing ameloblastoma margins. A case is presented in which margins are assessed with each of clinical assessment based on preoperative imaging, intra-operative specimen x-ray, intra-operative specimen computed tomography (CT) and definitive histology. Each modality is compared quantitatively. These results are compared to the literature through means of systematic review of current evidence.

**Results:**

A comparative study highlights the role for CT imaging over plain radiography. With no other comparative studies and a paucity of high level evidence establishing a role for intra-operative margin assessment in ameloblastoma in the literature, only level 4 evidence supporting the use of frozen section and specimen x-ray, and only one level 4 study assesses intra-operative CT.

**Conclusion:**

The current study suggests that intra-operative specimen CT offers an improvement over existing techniques in this role. While establishing a gold-standard will require higher level comparative studies, the use of intra-operative CT can facilitate accurate single-stage resection.

## Background

Ameloblastoma is a locally aggressive and destructive odontogentic tumor of either the maxilla or mandible, which is associated with recurrence rates of up to 90% if not completely excised [1]–[5]. As such, adequate surgical clearance is one of the key factors in effective treatment of ameloblastoma [6]. Despite this, there is little in the literature as to how to achieve this.

Anecdotally, the use of preoperative imaging to guide the location for excision margins has been the mainstay of operative approach [7]–[10], with definitive histology used for confirmation postoperatively. However, the need to decalcify the specimen can mean waits of up to 4 weeks for this confirmation. The use of specimen x-ray to confirm margins intra-operatively has thus been performed [1], [11]–[15], however plain x-ray has been shown to be inaccurate at matching histologic margins [12], [16]–[19].

While there are other modalities used to assess tumor margins intra-operatively, such as Moh’s micrographic surgery (surgical excision with intra-operative microscopic examination of surgical margins), these are not suitable for ameloblastoma due to the process of decalcification, and thus other techniques for intra-operative assessment of margins are warranted.

Advanced imaging technologies, such as computed tomography (CT) imaging and magnetic resonance imaging (MRI) have been sought as improvements over plain films, with CT shown to be of greater efficacy and accuracy than plain x-rays in diagnostic scans [20]–[23], and MRI similarly showing greater efficacy in diagnosis and assessment of ameloblastoma [22]. However the role of these modalities in intra-operative margin evaluation has not been established in the literature.

The current manuscript aims to systematically review the literature in order to establish the gold standard for intra-operative evaluation of margins in the surgical management of ameloblastoma, presenting an analysis of the level of evidence for each currently described technique. The role for CT scanning, through both clinical report and review of the literature, is also presented; to determine which form of intra-operative specimen imaging is the most accurate tool in assessing adequate surgical margins patients undergoing ameloblastoma resection?

**Figure 1:**
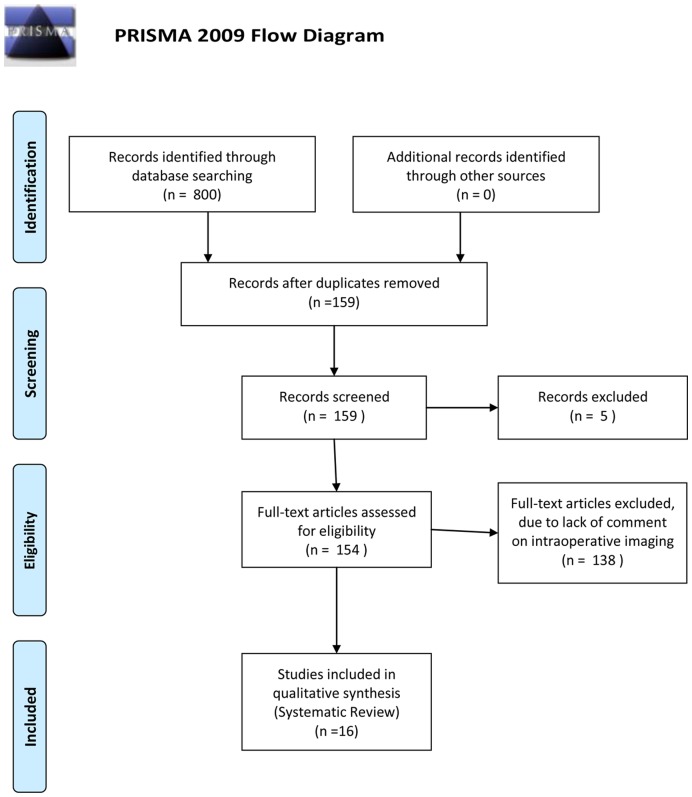
Citation attrition diagram documenting the systematic review search process as per PRISMA (Preferred Reporting Items for Systematic Reviews and Meta-Analyses).

**Figure 2:**
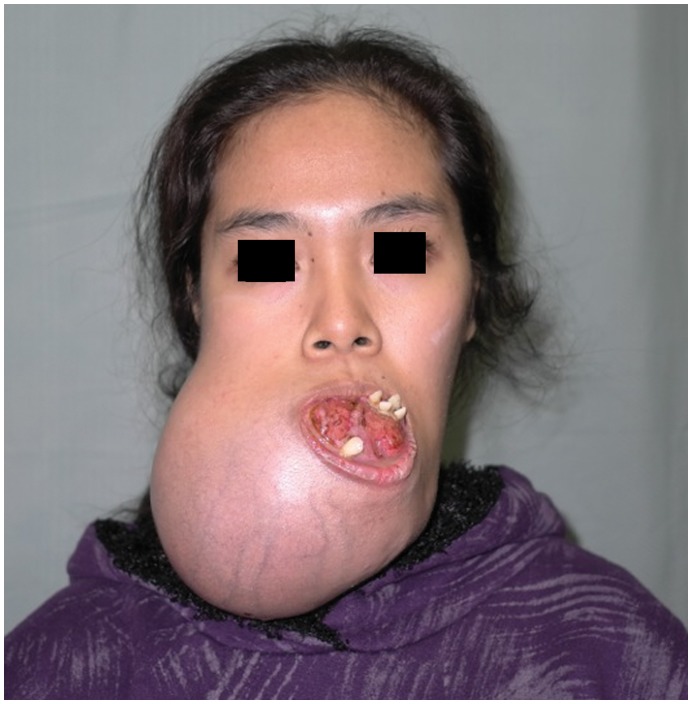
Preoperative photograph, demonstrating a large right mandibular ameloblastoma, causing an inability to eat and speak comfortably and severe cosmetic disfigurement. Reproduced with permission from: Fox C, Rozen WM, Ramakrishnan A, Baillieu C, Mirkazemi M, Leong J (2012) Microvascular Mandibular Reconstruction for Neglected Mandibular Tumours in the Third World: Bringing Humanitarian Aid Home. Plast Reconstr Surg. In Press.

**Figure 3:**
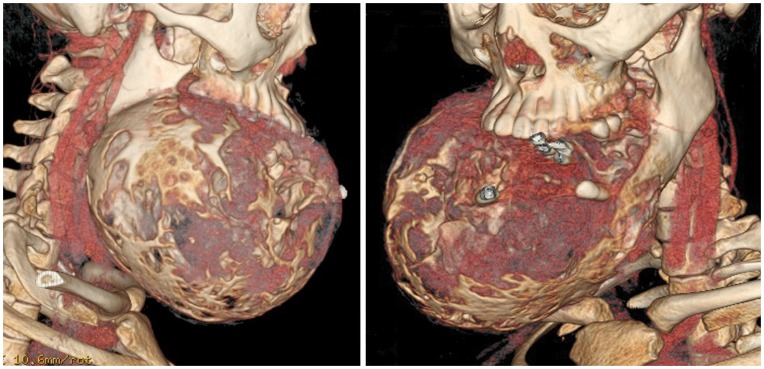
Preoperative computed tomographic (CT) imaging demonstrating the right mandibular ameloblastoma, with tumor dimensions measuring 13.8×11.2 cm in maximum axial dimension.

**Figure 4:**
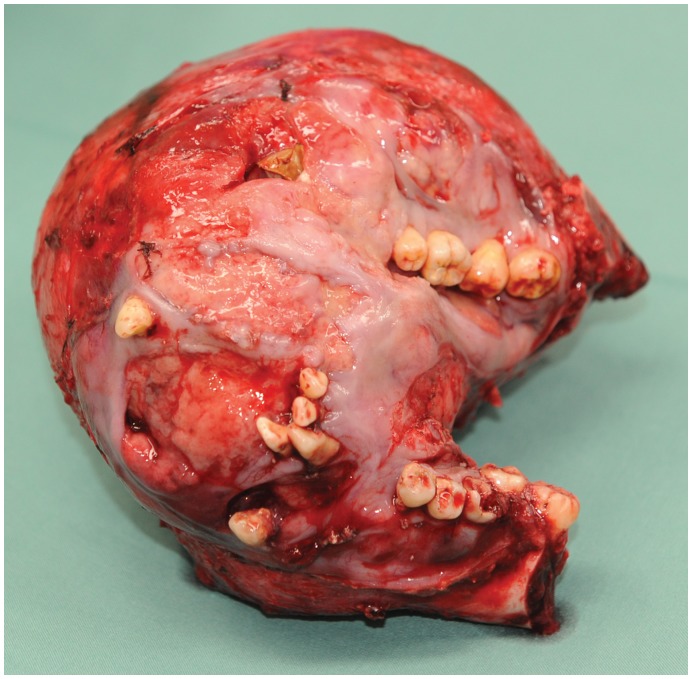
Intra-operative photograph of the ameloblastoma specimen after excision.

**Figure 5:**
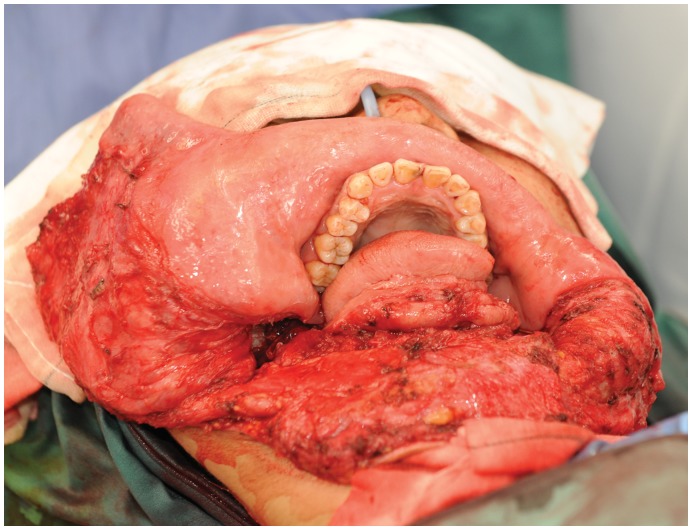
Intra-operative photograph of the patient after segmental mandibulectomy for ameloblastoma resection, highlighting the wide margins achieved, prior to any reconstruction.

## Methodology

The current study comprises the first reported comparative study of imaging modalities for assessing ameloblastoma margins, achieved through a systematic review of the current evidence. Currently no protocols exist to assist in the assessment of adequate ameloblastoma resection. This search was performed using PubMed, Medline, Cochrane databases, Web of Science and Google Scholar. Search terms included multiple combinations of ameloblastoma, radiograph, radiography, x-ray, specimen, imaging, operative, intra-operative, surgery, surgical, margin, frozen section, computed tomography, yielding between 2 and 210 results for specific search combinations. Inclusion criteria comprised English language studies on humans, and studies based on or including assessment of ameloblastoma surgical margins. Each of these studies was reviewed, with additional references identified through bibliographic linkage and included in the review. All references meeting inclusion criteria were independently assessed by a single author, with included studies restricted to 16. A PRISMA (Preferred Reporting Items for Systematic Reviews and Meta-Analyses) [24] flowchart for literature attrition is included (Figure 1). Bias risk was not specifically identified in these studies, however level of evidence was assessed formally according to CEBM (Centre for evidence Based Medicine) evidence level. The CEBM (http://www.cebm.net) attributes standardized levels of evidence, from level 1a (systematic review of randomised control trials) to level 5 (expert opinion), to any research paper. Each of the included studies was thus critically appraised based on their study design and content.

The literature findings were further assessed in the context of a case study, in which margins are assessed with each of clinical assessment based on preoperative imaging, intra-operative specimen x-ray, intra-operative specimen CT and definitive histology. Each modality was compared quantitatively. Ethical standards followed the provisions of the National Health and Medical Research Council and the Declaration of Helsinki in 1995. The subject gave informed consent and patient anonymity was preserved.

**Figure 6:**
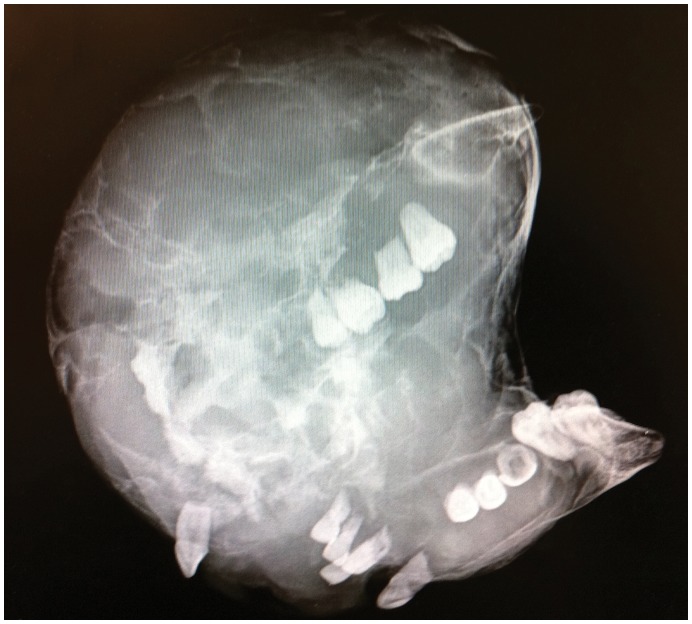
Intra-operative specimen radiograph with plain x-rays.

**Figure 7:**
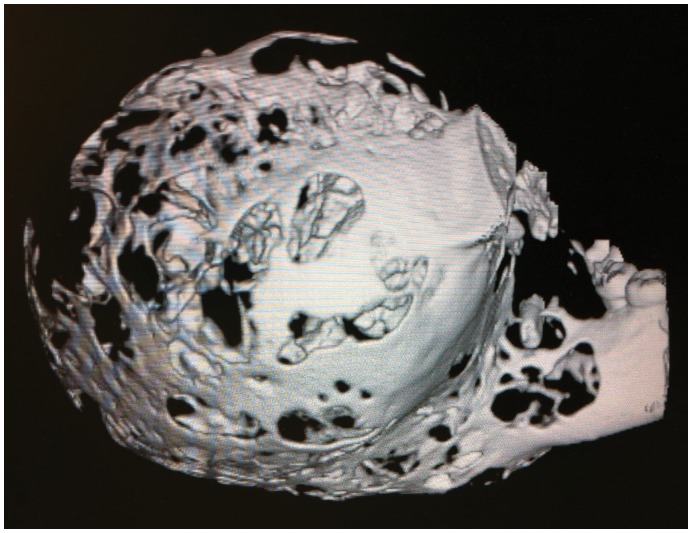
Intra-operative specimen computed tomogram (CT), with volumetric three-dimensional reconstruction image shown.

**Figure 8:**
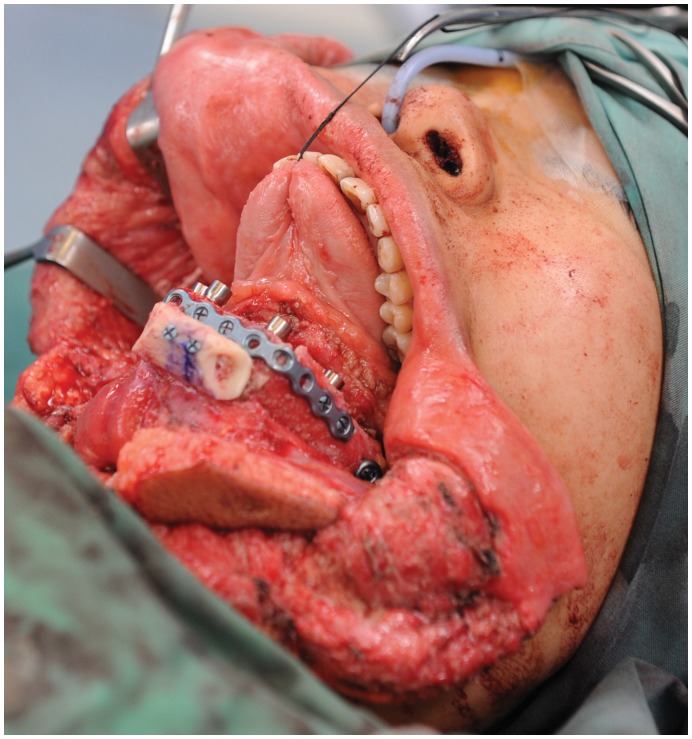
Post-resection mandible reconstructed with free fibula flap, contoured with two closing wedge osteotomies, osseointegrated dental implants placed primarily, and a genioplasty performed with fibula bone graft to recreate chin contour.

**Figure 9:**
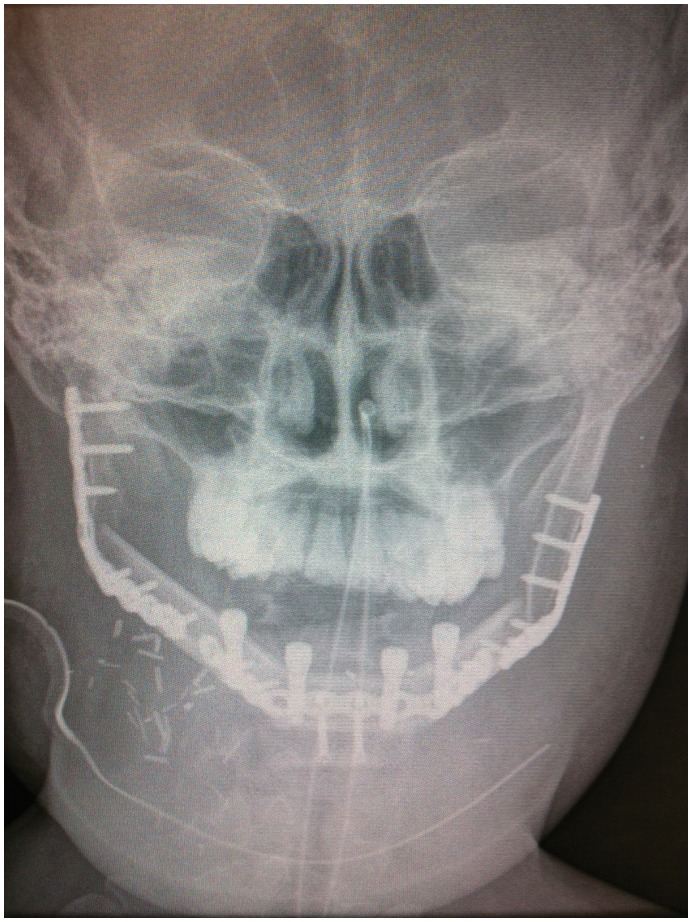
Postoperative x-ray, demonstrating good bony contour, with reconstruction plates used for mandibular fixation, screws used for genioplasty and osseointegrated dental implants inserted primarily.

**Figure 10:**
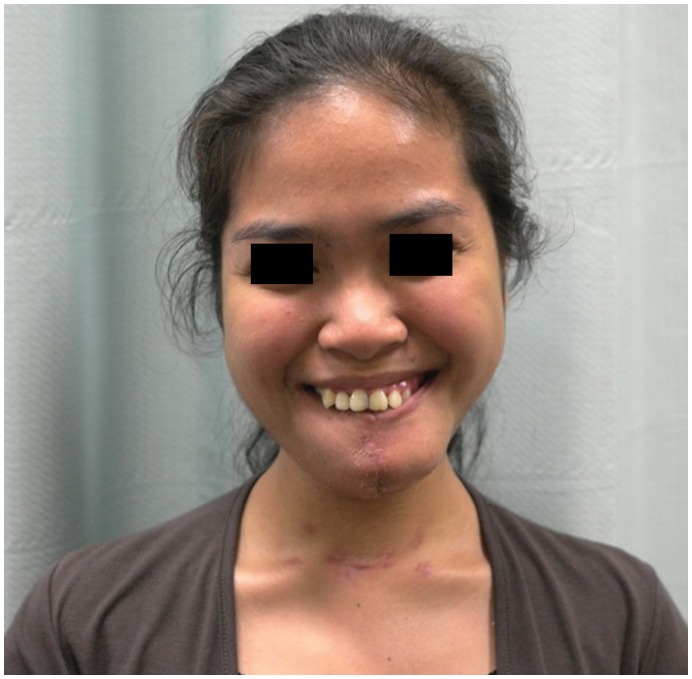
Postoperative photograph following resection and free fibula flap reconstruction, with only placement of a dental prosthesis outstanding to conclude her reconstruction. Postoperative smile highlights both the good cosmetic and functional outcome. Reproduced with permission from: Fox C, Rozen WM, Ramakrishnan A, Baillieu C, Mirkazemi M, Leong J (2012) Microvascular mandibular reconstruction for neglected mandibular tumours in the third world: Bringing humanitarian aid home. Plast Reconstr Surg. In Press.

**Figure 11:**
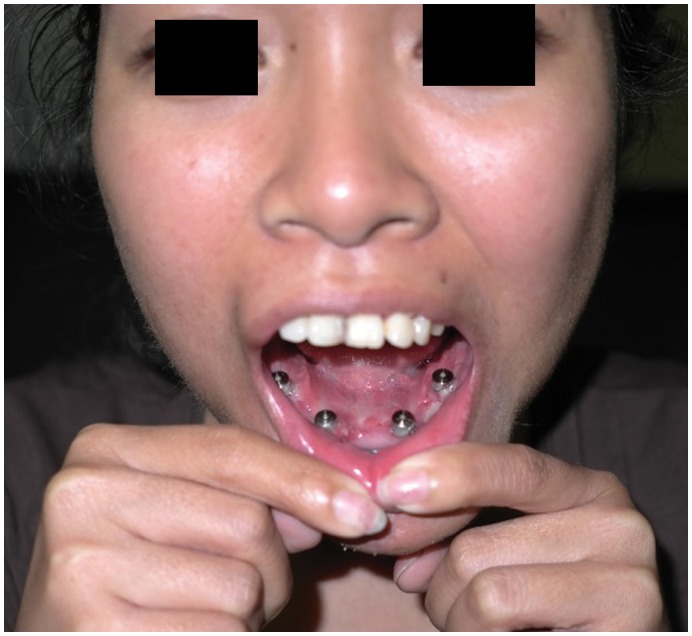
Postoperative photograph of the osseointegrated dental implants, awaiting placement of the definitive dental prosthesis.

## Results

### Literature Review

The systematic review of the literature identified 16 papers discussing imaging assessment of intra-operative margins in ameloblastoma resection. While the recent literature also discusses the use of frozen section to assess intra-operative margins, this did not form part of the systematic review but results were included for qualitative, comparative purposes. The reported methods for intra-operative margin assessment comprised plain specimen radiography [1], [5], [11]–[17], [25]–[30], frozen section (FS)[12], [15]–[17], [26], [31]–[39] and CT imaging [40]. No single study compared the use of intra-operative specimen X-ray to intra-operative CT.

### Intra-operative Specimen Radiography

The reported standard of practice has been to perform intra-operative specimen x-ray to grossly assess the adequacy of surgical margins, despite previous investigators reporting the potential for histologic extension of ameloblastoma beyond the apparent radiographic margins [12], [15], [25]–[27]. In most cases however, intra-operative x-ray has demonstrated the demarcation of typical unicystic or ‘soap-bubble’ appearance of ameloblastoma to normal bone. The false negative rates reported have been described as being due to the nature of tumor extension into bone, as expansion of the leading edge of the tumor erodes cortical bone and/or infiltrates through cancellous bone [5], [17], [28], [29]. The latter has widely been described as being difficult to detect on x-ray.

In determining the efficacy of intra-operative x-ray, the highest level of evidence achieved for establishing the role of intra-operative specimen x-ray is level 4, with a retrospective cohort study by Keszler et al [30] reviewing 162 ameloblastomas in a single oral pathology diagnostic centre over 35 years. This study utilized specimen x-ray in these cases with good utility, but did not compare to other techniques of margin assessment. Lower level studies (level 5), such as a single case presentation by Marx et al [16] described the mean extension into cancellous bone of tumor being 4.5 mm, with all results between 2.3–8 mm, beyond margins defined on specimen x-ray. These investigators suggest resection margins of 1 to 1.5 cm. Many other level 5 publications comment on the use of intra-operative specimen radiography; Cranin et al [14] and Cankurataran et al [13] describe the use of the specimen radiograph to visualize the extent of the cystic component in germ cell and unilocular ameloblastoma resection respectively; Imazawa et al [11] and Sham et al [1] performed specimen radiography on solid or mulitcystic ameloblastoma, but did not comment on use for assessment of margins; Carlson & Marx [12] and Black et al [15] described specimen radiography in solid or multicystic ameloblastomas as an opportunity to assess margins and remove additional bone to improve chance of cure [12] and to confirm margin adequacy [15].

No investigators compared intra-operative radiography to other modalities, nor did any comment specifically on their reliance of the intra-operative specimen radiography, with no authors describing changing the operative plan or performing further resection based on the results of the intra-operative specimen radiograph.

### Intra-operative Frozen Section

Intra-operative frozen section has been used for immediate sampling and assessment of tumor margins to ensure adequate resection of tumor, to provide ‘the surgeon significant guidance regarding the required extent of the surgical procedure’ [26], with the widely reported accuracy of FS approximating 95% [31], [32].

Again, there is no high level evidence to support the use of FS over other intra-operative techniques for margin assessment. Level 4 evidence was presented by Winther & Graem [31], who reviewed 4785 pathologic cases of intra-operative frozen section over a 1 year period to assess the accuracy of FS in diagnosis or adequacy of resection. These investigators identified concordance rates of 91.5% with definitive histology for overall accuracy, with a false-negative rate of 3.8%. Similarly, a large level 4 case series by Gephart et al [32] examined 90,538 cases, with an overall specimen concordance rate of 98.38% with the majority of discordance attributed to misinterpretation of the FS (31.8%), inaccurate sampling of the FS (30%) or gross sampling errors (31.4%).

In regard to the specific use of FS in ameloblastoma resection as a means to guiding surgical resection, many investigators have recommended the use of FS to assess soft tissue margins in several scenarios: either as a routine (all cases) [12], when clinical soft tissue involvement is evident clinically [15], [16], [32]–[35], or when underlying bony cortices are perforated clinically or on preoperative imaging [36]. In determining the efficacy of FS in these scenarios, Matsumara et al [37] presented a level 4 study of intra-operative FS and the use of rapid flow cytometry on 60 patients over a 3 year period. FS was used to determine surgical margins in 71.7% of cases with overall accuracy of FS found to be 98%. The use of flow cytometry assisted the investigators in distinguishing benign and malignant lesions. Another level 4 retrospective study by Guthrie et al [38] reported discordance rates of 46%, attributing inaccuracy to sampling error, insufficient tissue (15%) and inflammation (15%). A case report by Marx et al [16], highlighting the utility of FS identified positive intra-operative margins in ameloblastoma resection while performing routine FS of soft tissue margins, with one margin showing tumor invasion through tendon and adjacent periosteum prompting further bony resection.

Although, most commonly used to assess soft tissue margins, more recently FS has also been applied to assessment of tumor invasion into cancellous bone [12], [17]. A well constructed level 4 case series by Forrest et al [17] examined 61 surgical margins, where intra-operatively approximately 1 cm of cancellous bone was removed from the remaining stump of the mandible and was analysed with FS with comparisons made to definitive histology post de-calcification. A 98% concordance rate was established with sixty of sixty-one margins exactly correlated to definitive histology, with 100% specificity. Specifically, Forrest et al [17] identified positive cancellous margin in an ameloblastoma which prompting further resection and repeat FS. A negative second cancellous FS correctly correlated to the definitive histology, post de-calcification. Carlson and Marx [12] presented level 5 evidence, reviewing primary surgical management of ameloblastoma, recommending routine FS soft-tissue assessment during resection of ameloblastoma, as well as the use of FS on the medullary portion of the stump of bone in the tissue bed when radiological margins are less than 1 cm, rather than resecting additional bone.

In a level 4 case series of 84 ameloblastomas, addressing approach to treatment and length of follow up, Muller et al [39] found that even with the assistance of FS they were unable to prevent the recurrence of ameloblastoma in one particular case. However they did not discuss the use of FS in the other 83 reported cases.

### Computed Tomography

The routine use and efficacy of conventional multi-slice CT in the assessment of intra-operative margins for ameloblastoma resection has not yet been discussed in the literature. A recent case series assessing the utility of intra-operative CT in craniofacial bony specimens, was presented by Schaaf et al [40]. These investigators described the application of an experimental ‘flat panel volumetric’ CT (fpvCT) in assessing intra-operative bony margins for human craniofacial bone pathology, in 35 bony specimens, including 4 ameloblastomas, prior to intra-operative frozen section. Intra-operatively a fresh specimen fpvCT was performed with reconstructed images displayed on an Advantage Workstation. FS was performed with a 2D image projected on a screen next to the fpvCT image. The 3D fpvCT image was rotated to provide an identical image plane to that of the 2D FS plane. These images were compared. These investigators describe their results as analogous to fresh FS. FpvCT images were also compared to pre-operative multi-slice CT images. Shaaf et al concluded that for cortical and cancellous bone architecture fpvCT was able to provide a higher precision and more detail in fine bone structure and tumor borders than multi-slice CT, and were able to scan specimens up to 33×33×21 cm, with scanning time between 8 seconds and 1 minute.

This level 4 evidence is all that has been presented in the literature in support of advanced imaging technologies for intra-operative margin assessment.

### Comparative Study

A 24 year old female from the Philippines, presented with a large painless mass in right jaw, with a biopsy overseas having confirmed a right mandibular ameloblastoma (Figure 2). She had not sought treatment overseas, and a preoperative CT (Figure 3) highlighted a large, heterogenously enhancing destructive mass arising from the right side of the mandible, measuring 13.8×11.2 cm in maximal axial dimensions and 11.3 cm in maximal cranio-caudal length. The inferior extended to level of the sterno-clavicular joints. The imaging findings of a radiolucent, multicystic lesion with a ‘soap-bubble’ appearance were pathognomonic of ameloblastoma, with cortical expansion and thinning also seen. A wide excision of the right mandibular ameloblastoma was thus undertaken (Figures 4 and 5), with 1 cm resection margins planned intraoperatively based on direct clinical observation of the abnormal bone. These clinically assessed margins were evaluated with subsequent specimen imaging, described in detail below. An immediate mandibular reconstruction with an osseo-musculo-cutaneous free fibular flap, immediate osseointegrated dental implants and genioplasty was planned.

Intra-operative specimen X-ray (Figure 6) illustrated large soft tissue mass with multiple areas of internal ossification, ‘soap bubble appearance’ and teeth present, with portion of the the involved mandible noted with resection margins to normal bone of 0.75 cm on the right and 1.72 cm on the left. Intra-operative CT (Figure 7) was performed and reviewed by a radiologist in comparison to the pre-operative CT to assist in evaluating anatomical alignment of the mandible. Intra-operative CT showed a large bony and soft tissue specimen containing numerous cystic spaces and bony fragments with a thickened sclerotic appearance suggesting that this is likely the result of chronic remodeling/expansion rather than aggressive destruction. Resection margin of the tumor achieved margins of 0.71 cm on the right and 1.1 cm on the left (Figure 8). These findings were discussed intra-operatively, confirming clinically clear margins. No further excision was undertaken. Post-operative x-ray demonstrated good bony contour, with reconstruction plates used for mandibular fixation, screws used for genioplasty and osseointegrated dental implants inserted primarily (Figure 9). The patient had a good cosmetic and functional outcome (Figure 10), and with osseointegrated dental implants in-situ, is only awaiting placement of the definitive dental prosthesis to complete her reconstruction (Figure 11).

Definitive post-operative histology illustrated an expanded, greatly distorted mandible measuring 160×130×90 mm with fifteen teeth. The mandible included the body, angle and parts of the rami with a large mass centred in the mandible bone being more prominent on the right side, measuring about 13 cm medial to lateral, 12 cm anterior to posterior and 9 cm superior to inferior. The mandibular rami resection margins were clear of tumor, with left sided margins 1.5 cm clear and right sided margins 1 cm clear. The rim of gingival margins was also clear of tumor. As such, both imaging modalities compared favourably to definitive histology, while finer features of the CT results were felt to be more accurate than plain film radiology, and to contribute more to achieving clear margins (Table 1).

**Table 1:** Comparison of margins for intra-operative specimen x-ray, Computed Tomography and definitive post-operative histology.

Modality	Right Margin	Left Margin
Plain X-ray	0.75 cm	1.72 cm
Computed Tomography (CT)	0.71 cm	1.1 cm
Histology	1 cm	1.5 cm

The clinical report as described highlights the utility of the techniques presented within the literature review, and presents the first comparison of the radiographic options for intra-operative margin assessment.

## Discussion

Ameloblastoma is a rare, benign, but locally invasive odontogenic neoplasm originating from epithelial cells. Found mainly in the posterior body or angle of the mandible, these tumors may also be present anywhere in the mandible (80%) or the maxilla (20%) [1]. Ameloblastomas represent one percent of all oral tumors [41] and 11% of all odontogenic tumors [42]. Ameloblastomas affect men and women equally and most commonly occur in the 3^rd^ and 4^th^ decades of life [43]. They frequently present as asymptomatic intraoral swelling but may be associated with a variety of symptoms including pain, paresthesia and loose teeth [44], or occasionally found incidentally on routine dental x-rays. If untreated, ameloblastoma can result in severe facial deformity and they are associated with extensive local bone erosion and destruction [15].

Previous reports of the imaging of ameloblastoma has highlighted the plain x-ray appearance of ameloblastoma to include lytic and radiolucent lesions, depicted as unilocular, multicystic or having a ‘soap-bubble’ appearance [22], [45], [46], but this modality has been found to have false positive (40–50%) and false negative (5–7%) rates in the evaluation of mandibular tumor invasion [22]. Previous investigation has also shown that in addition to the characteristics described on x-ray, CT is able to evaluate the integrity of the cortex by visualizing expansion and thinning, define the extension of tumor into adjacent structures and the presence of soft tissue mass formation [22]. These images are created without superimposing anatomical structures and exhibit no magnification or distortion [23].

Current recommendations in the treatment of ameloblastoma is segmental resection, with at least a 1–2 cm margin [1], [12], [25]–[27], [47], [48], and immediate bony reconstruction [1], [49], [50]. Evaluation of the current literature has highlighted clinical benefit in performing intra-operative FS on cancellous bone to assess margins, with Forrest et al [17] showing concordance rates of 98% with definitive histology, and other level 4 evidence supporting this role. Analysis of soft tissue FS identified concordance rates of 61–98% [37], [38], with sampling error being the main factor in discordant results.

The use of fpvCT by Shaaf et al [40] provided images for assessment in identical planes to FS with analogous results. In comparison of fpvCT to pre-operative CT in mandibular ameloblastoma resection, fpvCT provided superior assessment of cancellous bone, which is of particular importance in determining the nature of extension of ameloblastoma.

In our case report, the first such study to compare intra-operative x-ray with CT, imaging compared favorably to post-operative histology. Plain x-ray, a 2-dimensional modality, was found to be difficult in the assessment of a larger specimen and has a lower rate of border identification; however it is quick and cost efficient, with good spatial resolution. Its particular benefit may lie in smaller specimens where the assessment of anatomical alignment is sought. Intra-operative CT was compared to the pre-operative CT to confirm anatomical position, yielding a higher degree of confidence in surgical margins. CT also had better contrast resolution, the ability to view the mass from all angles and the capacity to reconstruct images with both bone and soft tissue algorithms and 3-dimensional model creation. In this case, CT increased the degree of confidence of the left mandibular resection border measurement, which was not well seen on the x-ray due to the large mass size and distortion of normal anatomy, which required multi-planar assessment. The use of preoperative CT also complements preoperative CT scans, performed routinely in the diagnosis and assessment of ameloblastoma and other bony tumors of the facial skeleton [51]. These attributes highlight the improvements that CT can offer over plain X-ray and other existing techniques for intra-operative margin assessment. While CT was found to be superior to plain x-ray in this assessment, it is noteworthy that neither modality correlated exactly to the margins identified on definitive histology.

### Conclusions

Due to the high recurrence rate of ameloblastoma, measures are keenly sought to adequately assess intra-operative margins in order to ensure complete resection of this tumor. There is low level evidence supporting the use of each of FS, specimen x-ray and specimen CT, however no comparative studies have been undertaken. In our single-case comparative study, specimen CT was found to have improved contrast resolution, 3-dimensional spatial alignment and assessment of soft tissue involvement than plain x-ray, although neither technique was completely concordant with histologic margins; and therefore gives a higher degree of confidence in identifying surgical margins. The current study suggests that intra-operative clinical assessment and preoperative imaging are insufficient in the assessment of intra-operative margins for ameloblastoma, and that intra-operative specimen CT imaging offers an improvement over existing imaging techniques in this role. While establishing a gold-standard will require higher level comparative studies, the use of intra-operative CT can facilitate accurate single-stage resection.
